# The Impact of Differentiation on Cytotoxicity and Insulin Sensitivity in Streptozotocin Treated SH-SY5Y Cells

**DOI:** 10.1007/s11064-021-03269-2

**Published:** 2021-02-22

**Authors:** Fruzsina Bagaméry, Kamilla Varga, Kitti Kecsmár, István Vincze, Éva Szökő, Tamás Tábi

**Affiliations:** Department of Pharmacodynamics, Semmelweis University, Nagyvárad tér 4, 1089 Budapest, Hungary

**Keywords:** GLP-1 analogue, Glycogen-synthase kinase-3, Insulin resistance, SH-SY5Y cell line, Streptozotocin, Retinoic acid induced differentiation

## Abstract

Recently neuronal insulin resistance was suggested playing a role in Alzheimer’s disease. Streptozotocin (STZ) is commonly used to induce impairment in insulin metabolism. In our previous work on undifferentiated SH-SY5Y cells the compound exerted cytotoxicity without altering insulin sensitivity. Nevertheless, differentiation of the cells to a more mature neuron-like phenotype may considerably affect the significance of insulin signaling and its sensitivity to STZ. We aimed at studying the influence of STZ treatment on insulin signaling in SH-SY5Y cells differentiated by retinoic acid (RA). Cytotoxicity of STZ or low serum (LS) condition and protective effect of insulin were compared in RA differentiated SH-SY5Y cells. The effect of insulin and an incretin analogue, exendin-4 on insulin signaling was also examined by assessing glycogen synthase kinase-3 (GSK-3) phosphorylation. STZ was found less cytotoxic in the differentiated cells compared to our previous results in undifferentiated SH-SY5Y cells. The cytoprotective concentration of insulin was similar in the STZ and LS groups. However, the right-shifted concentration–response curve of insulin induced GSK-3 phosphorylation in STZ-treated differentiated cells is suggestive of the development of insulin resistance that was further confirmed by the insulin potentiating effect of exendin-4. Differentiation reduced the sensitivity of SH-SY5Y cells for the non-specific cytotoxicity of STZ and enhanced the relative significance of development of insulin resistance. The differentiated cells thus serve as a better model for studying the role of insulin signaling in neuronal survival. However, direct cytotoxicity of STZ also contributes to the cell death.

## Introduction

One consequence of the progressive society aging is the rapid rising in the prevalence of Alzheimer’s disease (AD) [1]. In the central nervous system insulin is not only responsible for metabolic regulation but neuroprotection and improving of cognitive processing are also among its major roles [2, 3]. It signals via the phosphatidylinositol-3 kinase (PI3K)-protein kinase B (Akt/PKB) pathway [4] that in turn phosphorylates and inactivates glycogen synthase kinase-3 (GSK-3) [5]. GSK-3 has two isoforms and both α and β were shown associated with neurodegenerative processes, namely they participate in the development of tauopathy and amyloidopathy [6–8]. Insulin resistance has been recently suggested to contribute to the development of AD and cognitive decline [2, 3, 9, 10] that may be explained by the inappropriate regulation of GSK-3 [11–14].

Improvement of central insulin sensitivity might serve as a therapeutic tool to fight against neuronal degeneration in AD. Incretin hormones, like glucagon-like peptide-1 (GLP-1) are among the suggested neuroprotective agents acting by reducing insulin resistance in the brain (for review: [10]). Analogues of GLP-1, like exendin-4 (exenatide, Ex-4) were recently introduced to the therapy of type 2 diabetes. Besides its beneficial peripheral effects, Ex-4 was suggested mediating neuroprotection by potentiating central insulin signaling [10, 15]. In in vitro studies on neural cell cultures its protective effects against various damaging conditions like oxidative stress [16, 17], hypoxia [18] or Aβ insult [16, 19] among others was revealed. Better understanding the role of insulin signaling in the central nervous system and its potential modulation by pharmacological tools like GLP-1 analogues therefore can facilitate the research of disease modifying therapy for AD.

Streptozotocin (STZ) treatment is one of the most established method for induction of impairment in the insulin system [20]. In in vivo experiments it can induce either type 1 or type 2 diabetes [21–23]. In addition, the compound was used as a diabetic neuropathic pain model because its administration results in the development of allodynia besides hyperglycemia [24, 25]. Furthermore, its local, intracerebroventricular (*icv.*) administration is also used to induce AD like brain damages characterized by elevated oxidative stress [26] and increased activity of GSK-3β [27, 28] among others.

STZ is also used in in vitro experiments to explore the cellular mechanisms that might contribute to cell death in AD. It was found to have concentration-dependent cytotoxic effect in various neural cell types [29–32]. Mitochondrial damage [29, 32, 33], elevated reactive oxygen species production [34] and higher rate of apoptosis [33–35] were observed. Its effect on insulin system was also examined showing reduced expression of insulin receptor substrate (IRS)-1 [36], altered GSK-3 phosphorylation [31, 34] and increased tau protein phosphorylation [35]. Additionally, insulin was found to improve STZ induced cytotoxicity [29, 34].

In our previous experiments we studied the effect of STZ on insulin sensitivity of undifferentiated SH-SY5Y neuroblastoma cells. It induced concentration- and time-dependent cytotoxicity that was concentration-dependently ameliorated by insulin with a potency similar to that seen in case of control damage induced by low serum medium (LS). Moreover, there was no significant difference in the insulin induced phosphorylation of GSK-3 between STZ and LS treatments indicating maintained insulin sensitivity in STZ treated undifferentiated SH-SY5Y cells [37]. Therefore, we concluded that STZ induces rather non-specific cytotoxicity to the undifferentiated cells without the development of significant insulin resistance, which might be explained by the high sensitivity of rapidly proliferating neuroblastoma cells to cytotoxic compounds like STZ [38].

SH-SY5Y cells can be differentiated to gain a more mature neuron-like phenotype. Although numerous differentiation methods exist, retinoic acid (RA) induced process is the most commonly used [39]. During differentiation among other altered cellular properties, decreased susceptibility to neurotoxins [40, 41] were observed. These findings indicate that differentiated neuroblastoma cells may serve as a more accurate translational model of human disease, therefore in our present study we aimed at examining the effect of STZ on cell viability and insulin signaling in RA differentiated SH-SY5Y cells.

## Materials and Methods

### Materials

Dulbecco’s Modified Eagle Medium/Nutrient Mixture F-12 (DMEM/F12) and fetal bovine serum (FBS) were obtained from Corning (Tewksbury, MA, USA) and Biosera (Nuaille, France), respectively. Stable glutamine and Minimum Essential Medium non-essential amino acids solutions were purchased from Pan Biotech (Aidenbach, Germany). Insulin, Ex-4, resazurin based cell viability kit (TOX-8), Triton X-100, Phosphatase inhibitor cocktail 2 were obtained from Sigma (St. Louis, MO, USA). STZ was purchased from Cayman Chemical Company (Ann Arbor, MI, USA) and was dissolved in citrate buffer (0.1 M, pH 4.5) immediately before adding to the medium. DuoSet IC, Phospho-GSK-3α/β (S21/S9) ELISA kit was provided by R&D Systems GmbH (Wiesbaden, Germany).

### Cell Culture, Differentiation and Treatment

Human neuroblastoma SH-SY5Y cells (ECACC, UK) were seeded to 10 cm Petri dishes (4 × 10^5^ cells/dish) or 24-well plates (2 × 10^4^ cell/well) and were cultured in DMEM/F12 containing 3.15 g/L glucose, 10% FBS, 1% stable glutamine and antibiotics. For induction of differentiation 50 µM RA was added to the culture medium on day 0 for 5 days. Culture medium was refreshed on the third day of differentiation.

Resazurin reduction cell viability assays were carried out in 24-well plates. After the 5-day-long differentiation the medium was changed to LS one that contained 1% FBS and treated by various concentrations of STZ (0.3, 1, 3, 5, 7, 10 mM). In some experiments the cells were simultaneously treated by insulin (10, 30, 100, 300, 1000, 3000 nM). The medium was refreshed daily, and insulin treatment was repeated alongside for up to 3 days. To determine the alteration of cell viability after STZ treatment the results were compared to the control damage, LS group. Furthermore, to assess the protective effect of insulin the rate of resazurin reducing activity in insulin treated or damage-only (STZ or LS alone) groups were analyzed.

For determination of GSK-3 phosphorylation the cells were treated by LS medium and 5 mM STZ after the 5-day differentiation. Twenty-four hours later the cells were treated with insulin (10, 30, 100, 300, 1000, 3000 nM) for 30 min then harvested in ice cold lysis buffer (1 mM EDTA, 0.5% Triton X-100, 6 M urea in PBS) containing Phosphatase inhibitor cocktail 2 and assayed by ELISA.

In some experiments, after the 5-day differentiation the cells were pretreated with 100 nM Ex-4 in LS medium for 1 h followed by addition of 5 mM STZ. Twenty-four hours later the cells were treated with insulin (100 nM) in the presence or absence of Ex-4 (100 nM) for 30 min then harvested in Phosphatase inhibitor cocktail 2 containing ice cold lysis buffer.

The concentration of 5 mM STZ and 100 nM insulin were chosen according to the results of our cell viability assays as these induced a low but significant cellular damage and a significant cytoprotection, respectively. The concentration of Ex-4 was chosen on the basis of the literature.

### Resazurin Reduction Cell Viability Assay

Resazurin reduction was assessed according to the manufacturer’s instructions. Briefly, after 24, 48 and 72 h STZ treatment the medium was replaced by fresh medium containing resazurin (0.015 mg/ml final concentration) and incubated for 4 h at 37 °C. The fluorescence of resorufin formed was measured by Fluoroskan Ascent FL microplate fluorimeter (Thermo Fisher Scientific, Waltham, MA USA) at 530 and 590 nm excitation and emission wavelengths, respectively. To characterize the cytoprotective effect of insulin, difference of resorufin fluorescence (RFU) between untreated and damaged cells was determined. Protection of each treatment was expressed as percent improvement in fluorescence:

1 $${\text{Protection}}\;(\% ) = 100 \times ({\text{RFU}}_{{{\text{treated}}}} - {\text{RFU}}_{{{\text{damaged}}}} )/({\text{RFU}}_{{{\text{undamaged}}}} - {\text{RFU}}_{{{\text{damaged}}}} )$$

### ELISA Measurement

GSK-3 phosphorylation was measured according to the manufacturer’s instructions with some modifications. Briefly 100 µL cell lysate was added to the ELISA plate and incubated overnight at 4 °C. After washing the plate was probed by detection antibody for 4 h at room temperature. The results were corrected by the protein content of the lysates measured by Bradford’s method [42].

### Statistics

Data are expressed as mean ± standard error of the mean of at least 3 parallel measurements. One-way ANOVA was used for data analysis followed by Dunnett’s post-hoc test for multiple comparisons. Concentration–response curves were constructed by non-linear regression method and the resulting estimates of Emax and EC50 were compared by F test. Corrected p < 0.05 was considered statistically significant. Data were analyzed by Prism 8 software (GraphPad Software Inc., La Jolla, CA, USA).

## Results

### STZ Induced Cytotoxicity on Differentiated SH-SY5Y Cells

The effect of STZ on cell viability was compared to that of LS condition on RA-differentiated SH-SY5Y cells exposed to 0.3, 1, 3, 5, 7, 10 mM STZ in LS medium. Resazurin reduction assay was carried out after 24, 48 and 72 h.

**Fig. 1:**
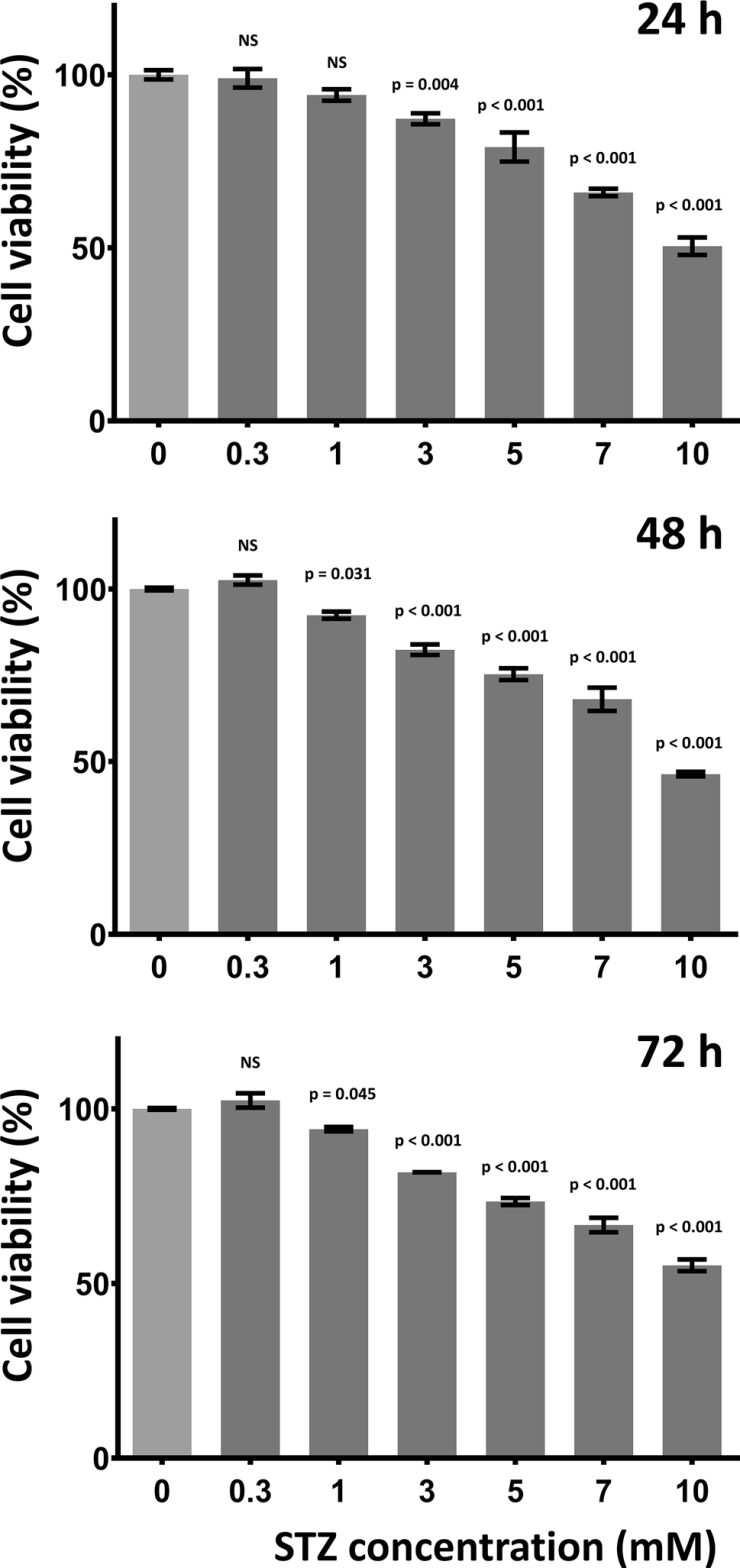
STZ concentration-dependently decreased cell viability compared to the cells treated with LS medium alone. P values from one-way ANOVA and Dunnett’s post hoc test compared to respective LS condition (0 mM STZ) are indicated, *NS* non-significant

Compared to the cytotoxic effect of LS treatment STZ further decreased the number of the metabolically active cells in a concentration-dependent manner. Lower concentrations, 0.3, 1 mM of STZ caused non-significant or mild decrease in cell viability, respectively, while higher concentrations (7, 10 mM) induced considerable decline in the number of viable cells. A significant but still moderate cytotoxicity could be observed after 3 or 5 mM STZ treatment indicating the eligibility of both concentrations to model the chronic neurodegenerative processes. For the further experiments 5 mM STZ treatment was chosen, compared to the LS condition it decreased the cell viability to 79.1%, 75.3% and 73.5% after 24, 48 and 72 h, respectively (Fig. 1).

### Insulin was Cytoprotective in LS and STZ Treated Cells

The impact of insulin was studied on both LS and 5 mM STZ induced cytotoxicity. Cells were treated by various concentrations of insulin (0, 10, 30, 100, 300, 1000, 3000 nM) and their viability was estimated after 24, 48 and 72 h by using resazurin assay.

**Fig. 2:**
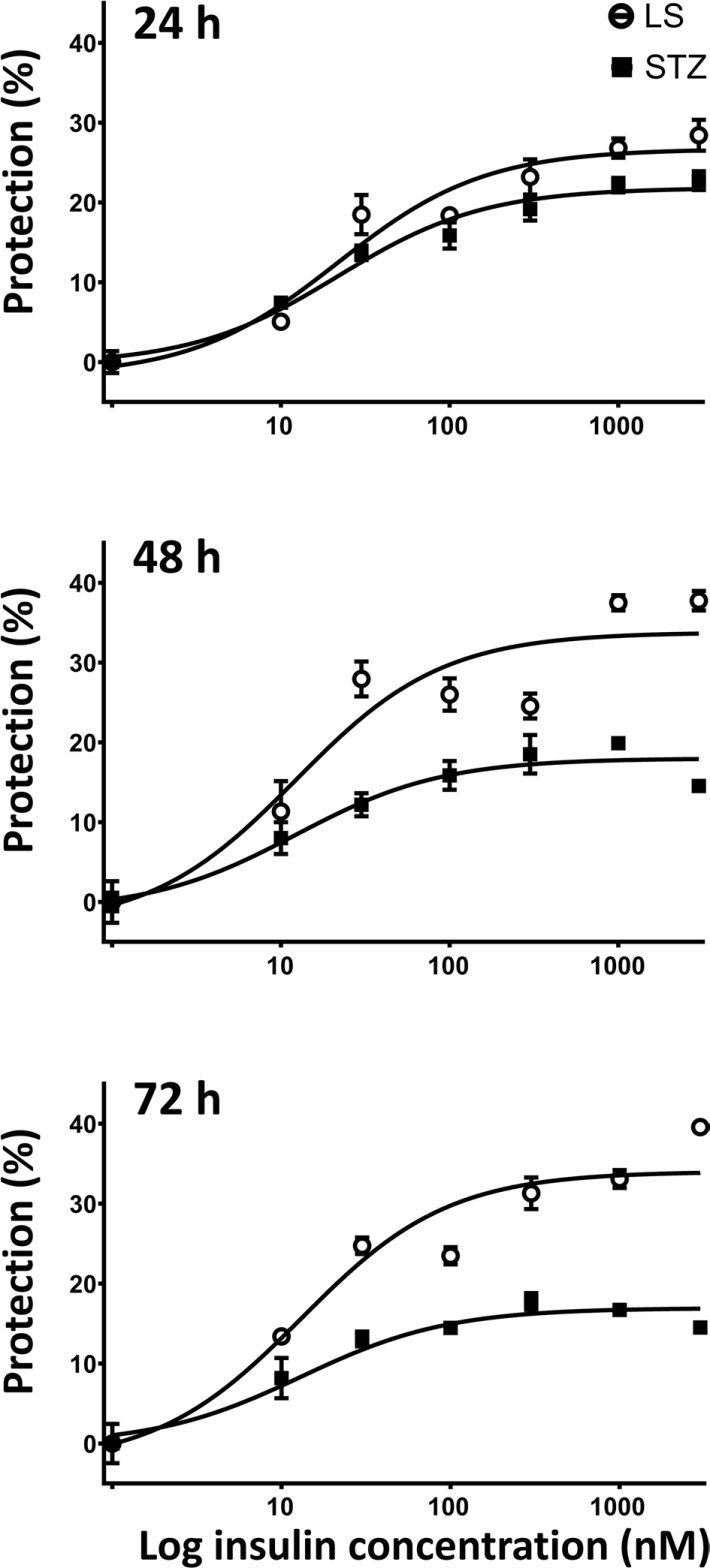
Insulin concentration- and time-dependently improved the cell viability. The percent protection against the damage in both LS or 5 mM STZ treated groups after 1, 2 and 3 days are shown. Curves were fitted by non-linear regression

In both LS and STZ treated cells insulin exerted concentration-dependent protective effect. In case of LS treatment insulin increased cell viability by 25–40%, which was significantly higher than seen in case of STZ treated cells where the maximal achieved protection was between 10–25% compared to the respective damage alone. The potency of insulin, however, was similar in both groups (Fig. 2).

### Insulin Induced GSK-3 Phosphorylation was Altered in STZ Treated Cells

**Fig. 3:**
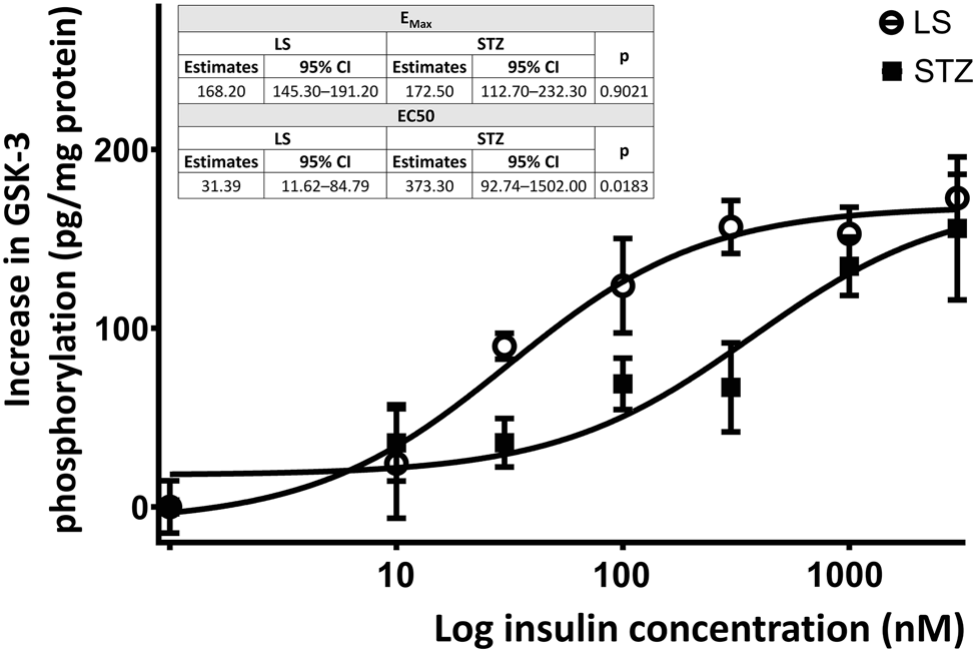
Insulin concentration-dependently increased the level of GSK-3 phosphorylation in LS or 5 mM STZ treated cells after 24 h with lower potency in the STZ group. Estimated EC50 and Emax values are shown in the insert. Insulin stimulation was used for 30 min before harvesting the cells for measurement of GSK-3 phosphorylation. Curves were fitted by non-linear regression estimates of EC50 and Emax were compared by F test

Insulin concentration-dependently elevated the level of phosphorylated GSK-3 in both LS and STZ treated cells. While the efficacy of insulin was similar, its potency was significantly different in the two groups. In the STZ treated cells the effective concentration of insulin was significantly higher as its dose response curve shifted to the right (Fig. 3). These data indicate the development of insulin resistance in the STZ treated cells compared to the LS group.

### Ex-4 Pretreatment Augmented the Insulin-Induced GSK-3 Phosphorylation in STZ Treated Cells

**Fig. 4:**
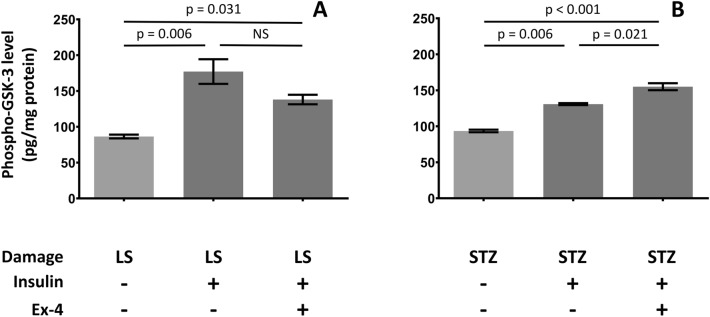
Ex-4 enhanced insulin induced GSK-3 phosphorylation only in STZ treated cells. Cells were pretreated with 100 nM of Ex-4 for 1 h before LS (**a**) or 5 mM STZ (**b**) treatment. After 24 h 100 nM insulin induced GSK-3 phosphorylation was measured by ELISA in the presence or absence of simultaneous 100 nM Ex-4 treatment. P values from one-way ANOVA and Dunnett’s post hoc test compared to respective damage conditions are indicated, NS: non-significant

Pretreatment with 100 nM Ex-4 for 24 h had also a different impact on the insulin-induced GSK-3 phosphorylation in LS and STZ treated cells. In the LS group Ex-4 pretreatment did not alter significantly the 100 nM insulin induced phosphorylation of GSK-3. However, in the STZ treated cells it significantly potentiated the effect of 100 nM insulin on GSK-3 phosphorylation (Fig. 4) confirming the impaired insulin sensitivity in response to STZ treatment.

## Discussion

Recently it has been suggested that impairment in brain insulin sensitivity can be an important factor in the complex pathomechanism of AD [28]. Some medications, such as intranasal insulin or GLP-1 analogues, like Ex-4 were shown to ameliorate the symptoms of the disease indicating that insulin signaling can be a potential medicinal target for treatment of AD [10, 15, 43, 44]. Numerous preclinical studies aim at investigating the precise mechanism of the disorder including the role of insulin signaling to facilitate the development of potential neuroprotective therapies.

In our previous in vitro study, we examined how insulin sensitivity of the undifferentiated SH-SY5Y neuroblastoma cells is influenced by STZ treatment. It was found to be suitable to induce a mild, gradual damage resembling to processes in chronic neurodegenerative disorders, however development of insulin resistance could not be observed [37].

SH-SY5Y cell line is also commonly used for neurochemical studies after differentiation. Among the various protocols the most commonly used RA [39] was chosen to promote mature neuron-like phenotype. Differentiation was induced using 50 µM RA for 5 days according to Jahn et al. [45] and the STZ-induced cytotoxicity and alteration in insulin signaling was studied on these differentiated cells.

Similarly to our previous study LS condition was chosen as control damage to diminish the effect of uncertain insulin concentration of FBS and as it is unlikely that insulin resistance develops due to reduced serum content of the medium [26, 46]. Previously in undifferentiated cells we found that 1 mM STZ caused a gradual time-dependent cellular damage [37], however this low concentration of the compound barely decreased the cell viability compared to the LS medium in the RA differentiated cells. To induce a low grade but reliable damage of these cells 3–5 mM STZ was needed. These findings are in accordance with the results of previous studies using similar undifferentiated cells like SK-N-MC human neuroblastoma cells or N2A mouse neuronal cell line [31, 33]. However, Isaev et al. reported that in immature cerebellar granule cells 3–4 mM STZ induced significant decrease in cell viability, while the mature cultures were hardly sensitive to this concentration [30] suggesting that not only cell type but its maturity is also a key factor in the sensitivity to neurotoxins. In addition, numerous papers reported that RA-induced differentiation of SH-SY5Y cells decreased their susceptibility to various noxious stimuli. Tieu et al. found that RA-differentiated cells were less vulnerable against various cytotoxic insults such as cisplatin, 5-flurouracil, 6-hydroxydopamine and γ-radiation. They explained their results by decreased expression of p53 tumor suppressor gene and the increased antiapoptotic Bcl-2 gene expression [41]. Similar results were reported by Cheung et al. as they found that RA-differentiated SH-SY5Y cell were less sensitive to 6-hydroxydopamine and 1-methyl-4-phenylpyridinium (MPP^+^) induced neurotoxicity [40]. In their experiments enhanced activity of neural survival signaling, i.e. increased PI3K/Akt activity was observed which was also reported by other groups [39, 47–49].

In previous studies cell damage induced by STZ was also found associated with impaired glucose metabolism and insulin signaling, such as reduction in the expression of glucose transporters in N2A neuronal and C6 glial cell lines [33], and that of insulin receptors [34] along with decreased phosphorylation of IRS-1 in SH-SY5Y cells [36] and dysregulation of GSK-3 activity in SK-N-MC cells [31]. In our previous study on undifferentiated SH-SY5Y cells STZ-induced cellular damage was concentration-dependently attenuated by the concurrent insulin treatment that is accompanied by improved GSK-3 phosphorylation. However, no difference in the potency of insulin was seen compared to the control LS induced damage [37].

In our present study the effect of insulin in LS and STZ treated RA-differentiated SH-SY5Y cells was compared. Insulin concentration- and time-dependently improved the cell viability. However, it was considerably less effective compared to the undifferentiated cells as the maximum protective effect was only 10–25% and 25–40% in the STZ and LS treated groups, respectively. Our present results thus indicate a significant alteration in insulin sensitivity during differentiation. Even though considerably different maximum protective effect of insulin was found in the LS and STZ treated differentiated cells the EC50 values of insulin for improving cell viability did not differ significantly, suggesting that STZ treatment did not alter the ability of insulin to promote neuronal survival even in RA-differentiated neuroblastoma cells. In both cases the efficacy of insulin was higher in LS groups, which could be explained by the more extensive damage induced by STZ.

GSK-3 was chosen to assess the activity of insulin signaling, as it is known to have an important role in neurodegenerative processes. The enzyme is regulated by various pathways, not exclusively by insulin, although its insulin induced phosphorylation can be suitable to examine the potential development of insulin resistance. We found that insulin concentration-dependently elevated GSK-3 phosphorylation in both LS and STZ treated cells, but its dose–response curve significantly shifted to the right in the STZ treated group indicating reduced insulin sensitivity of the STZ treated cells. This is in accordance with the findings of in vivo studies that showed STZ treatment induced insulin resistance both in the periphery [20] and the central nervous system [27, 28]. Since in our previous works on undifferentiated SH-SY5Y cells development of insulin resistance could not be observed [37], we can assume that the differentiation may induce alterations in the insulin signaling of the cells, that can make them more sensitive to the specific effect of STZ while its non-specific cytotoxic action was lower.

To further verify the impairment of insulin signaling in the STZ treated cells the effect of an incretin analogue, Ex-4 was also studied. Ex-4 was reported to improve neuronal insulin resistance both in vivo [50] and in vitro [15] experiments by preventing pathological phosphorylation of IRS-1, which interferes with its function to activate the downstream signaling cascade of insulin. The protective effect of Ex-4 is likely mediated by activation of GLP-1 receptors [15]. Ex-4 is frequently used in cell cultures as a potential neuroprotective agent. The compound prevented the damage induced by hydrogen peroxide [16, 17], Aβ [16, 19], staurosporin [17], glutamate [51, 52] in various neural cultures in 10^–7^ M concentration range.

In our present experiments pretreatment of cells with 100 nM Ex-4, a concentration that proved to be effective in various neuronal models [16–19, 51] significantly improved the insulin induced GSK-3 phosphorylation in STZ treated cells, while a similar improvement could not be observed in the LS group. This also indicates and confirms the presence of insulin resistance in the STZ treated differentiated SH-SY5Y cells.

To the best of our knowledge, we are first to present a suitable model to induce neuronal insulin resistant state in the frequently used SH-SY5Y neuronal cell line using STZ. However; limitations of our proposed model involve that apart from the development of insulin resistance in the differentiated cells the presence of considerable non-specific cytotoxicity of the compound must be taken into consideration too. Also, SH-SY5Y cells are not directly differentiated toward cholinergic phenotype and the role of other cell types of central nervous system too, such as microglias, oligodendrocytes and astroglias are not involved in our study. Therefore a possible improvement of our model may be altering the differentiation method e.g. directing it towards a more specific cholinergic phenotype [53] or using three-dimensional culture of SH-SY5Y cells to gain neurons with similar morphology and biochemistry like human neurons [54].

## Conclusion

Based on our previous [37] and present findings we can conclude that STZ treatment has distinct effect on the undifferentiated and RA-differentiated SH-SY5Y cells. Differentiation reduces the susceptibility of the cells for the direct cytotoxic effect of STZ and induces alterations that makes them more prone to its effect inducing insulin resistance. The STZ-treated differentiated SH-SY5Y cells thus are promising tools for studying the alterations in neuronal insulin signaling.

## Data Availability

The datasets generated during and/or analysed during the current study are available from the corresponding author on reasonable request.

## Abbreviations

AD: Alzheimer’s disease
Akt/PKB: Protein kinase B
Aβ: Amyloid beta
CREB: CAMP response element-binding protein
DMEM/F12: Dulbecco's Modified Eagle Medium/Nutrient Mixture F-12
Ex-4: Exendine-4
FBS: Fetal bovine serum
GLP-1: Glucagon-like peptide-1
GLUT: Glucose transporter
GSK-3: Glycogen synthase kinase-3
Icv: Intracerebroventricular
IRS: Insulin receptor substrate
LS: Low serum
MPP^+^: 1-Methyl-4-phenylpyridinium
PI3K: Phosphatidylinositol-3 kinase
RA: Retinoic acid
STZ: Streptozotocin
